# “Guilt by Association” Is the Exception Rather Than the Rule in Gene Networks

**DOI:** 10.1371/journal.pcbi.1002444

**Published:** 2012-03-29

**Authors:** Jesse Gillis, Paul Pavlidis

**Affiliations:** Centre for High-Throughput Biology and Department of Psychiatry, University of British Columbia, Vancouver, British Colombia, Canada; University of Chicago, United States of America

## Abstract

Gene networks are commonly interpreted as encoding functional information in their connections. An extensively validated principle called guilt by association states that genes which are associated or interacting are more likely to share function. Guilt by association provides the central top-down principle for analyzing gene networks in functional terms or assessing their quality in encoding functional information. In this work, we show that functional information within gene networks is typically concentrated in only a very few interactions whose properties cannot be reliably related to the rest of the network. In effect, the apparent encoding of function within networks has been largely driven by outliers whose behaviour cannot even be generalized to individual genes, let alone to the network at large. While experimentalist-driven analysis of interactions may use prior expert knowledge to focus on the small fraction of critically important data, large-scale computational analyses have typically assumed that high-performance cross-validation in a network is due to a generalizable encoding of function. Because we find that gene function is not systemically encoded in networks, but dependent on specific and critical interactions, we conclude it is necessary to focus on the details of how networks encode function and what information computational analyses use to extract functional meaning. We explore a number of consequences of this and find that network structure itself provides clues as to which connections are critical and that systemic properties, such as scale-free-like behaviour, do not map onto the functional connectivity within networks.

## Author Summary

The analysis of gene function and gene networks is a major theme of post-genome biomedical research. Historically, many attempts to understand gene function leverage a biological principle known as “guilt by association” (GBA). GBA states that genes with related functions tend to share properties such as genetic or physical interactions. In the past ten years, GBA has been scaled up for application to large gene networks, becoming a favored way to grapple with the complex interdependencies of gene functions in the face of floods of genomics and proteomics data. However, there is a growing realization that scaled-up GBA is not a panacea. In this study, we report a precise identification of the limits of GBA and show that it cannot provide a way to understand gene networks in a way that is simultaneously general and useful. Our findings indicate that the assumptions underlying the high-throughput use of gene networks to interpret function are fundamentally flawed, with wide-ranging implications for the interpretation of genome-wide data.

## Introduction

It is widely thought that to understand gene function, genes must be studied in the context of networks. Concurrent with this appreciation of complexity – and partially driven by it – the quantity of data available has grown enormously, especially for networks of interactions among genes or their products. Such networks can consist of millions of interactions across tens of thousands of genes, derived from protein binding assays [1]–[4], RNA coexpression analysis [5]–[7] and other methods [8]–[11]. In systems biology, there is enormous interest in using high-throughput approaches to systematically glean information from these networks (e.g., [12]–[15]). Information from such networks is now embedded in numerous studies and tools used by molecular biologists (e.g., [16], [17]), typically in combination with codifications of gene function exemplified by the Gene Ontology [18]. If one agrees that the function of a gene is partially a property determined by its context or relationships in the network, assessing the functional role of any given gene is challenging, as in principle one must consider all the interactions of the gene, in the context of the network.

Biologists have dealt with these challenges in part by leveraging the biological principle commonly referred to as “guilt by association” (GBA). GBA states that genes with related function tend to be protein interaction partners or share features such as expression patterns [19]. While not always referred to by name, GBA is a concept used extremely commonly in biology and which underlies a key way in which gene function is analyzed and discovered, whether on a gene-by-gene basis or using high-throughput methods. For example, an experimentalist who identifies a protein interaction infers a functional relationship between the proteins. Similarly two genes which interact genetically can be inferred to play roles in a common process leading to the phenotype [20]. This basic biological principle has been exploited by computational biologists as a method for assigning function in general, using machine learning approaches [21], [22]. This is made possible by the development of large interaction networks, often created by aggregating numerous isolated reports of associations as well as from high-throughput data sets. It has been repeatedly shown that in such networks there is a very statistically significant relationship between, for example, shared Gene Ontology annotations and network edges. Indeed, this relationship has even been used to “correct” networks so they are more highly aligned with GO annotations [23], , on the assumption that parts of the network that do not align with known function are more likely to be mistaken. Tremendous effort has gone into improving computational GBA approaches for the purpose of predicting function [25]–[32]. However, the number of biologically proven predictions based on such high-throughput approaches is still small and the promise of GBA as a general unbiased method for filling in unknown gene function has not come to fruition. In addition to their use in interpreting or inferring gene function, GBA approaches are also commonly used to assess the quality of networks, under the assumption that a high-quality network should map well onto known gene function information (see, for example, [33], [34]).

In computational applications of GBA, “performance” is usually assessed using cross-validation, in which known functions are masked from part of the network and the ability to recover the information is measured. A common metric is the precision with which genes sharing a function preferentially connect to one another [13], [25]; readers unfamiliar with prediction assessment methods are also referred to [35] and Text S1 (section 1). Built into this approach is the key assumption that GBA performance allows one to make statements about the network as a whole.

Gene function is not the only way in which networks are assessed. Another popular approach is to examine structural properties of the network, such as the distribution of node degrees in the network (number of associations per gene). It has been observed that many biological networks show “scale-free-like” behaviour (as evidenced by a power-law distribution of node degrees), or other related characteristics resulting in a heavy-tailed distribution of node degrees [36]. Similar to the situation for gene function, it is thought that a sign of high network quality is a power-law distribution of node degrees and some authors have even used this as a criterion for refining networks, on the assumption that data which conflicts with a power-law distribution is low-quality [37], [38]. The relationship between such properties and GBA has not been well-explored. While the significance of being scale-free is the subject of some debate [39], it is still commonly assumed that it reflects some more fundamental “biological relevance” of a network and contributes to the function of the network (and thus can be thought of “encoding functionality”). This paper represents an attempt to assess these types of assumptions, and in doing so derive some general principles about how function is “encoded” in current gene networks.

Previously, we showed that gene function can be predicted from networks without using “guilt”. We observed that a trivial ranking of genes by their node degrees results in surprisingly good GBA performance; about one-half of performance could be attributed entirely to node degree effects [35]. Node degree is predictive because genes that have high node degree tend to have many functions (e.g. GO terms; we call such genes “highly multifunctional”). Thus for any given prediction task, algorithms that assign any given function to high node-degree genes are rewarded by good performance without using information on which genes are associated with which. More concretely, when studying any biological process, simply assuming P53 (for example) is implicated will go a surprisingly long way, and networks encode this completely generic information in their node degree.

In this paper, we show that multifunctionality has a second effect on the interpretation of gene networks, and one that has especially serious implications for the interpretation and utility of GBA, and more generally for current assumptions about the how networks encode function. We focus on the identification of small numbers of connections between multifunctional genes, representing “exceptional edges” that concentrate functional information in a small part of the network. We show that networks of millions of edges can be reduced in size by four orders of magnitude while still retaining much of the functional information. We go on to show that this effect guarantees that cross-validation performance of GBA as currently conceived is a useless measure of generalizability with respect to the ability to extract novel information. Further, because information about biological function is not encoded in the network systemically, the edges that do encode function may not overlap with those generating “important” network-level properties, such as whether the network is scale-free. We determine that as currently formulated, gene function information is not distributed in the network as is commonly assumed. Instead, almost all existing functional information is encoded either in a tiny number of edges involving only a handful of genes, or not at all. We conclude that computational attempts to scale up and automate GBA have failed to capture the essential elements that made it effective on a case-by-case basis.

## Results

A key concept for our work is cross-validation, which is the means by which it is inferred that gene function can be predicted. In cross-validation, given one function of interest (for example, “inhibition of apoptosis”) and some genes which are already known to have that function (a “gold standard”), the function of some of those genes is masked (“held-out”). While there are some nuances as to how this is arranged, in general the investigator observes whether the algorithm can correctly assign function to the held-out set, using the remaining genes as a training set (and likewise that the function is not inappropriately assigned to genes considered negative examples). This procedure is repeated using different subsets of the data as training examples; each trial is called a “split”, referring to the division of the data into training and testing examples. In the analysis of any given split, genes which are “connected to” a training example are inferred to have the function. The definition of “connected to” is algorithm-dependent, but in a naïve approach this can be taken literally. Importantly, cross-validation only evaluates whether a function can be correctly predicted; it does not provide new predictions. This is the “generalization” problem: cross-validation is only useful to the extent to which it provides a good estimate of the accuracy of novel predictions. This is essential if one wants to predict gene function, as opposed to merely test algorithms. We will explore the problem of generalization by dissecting what part of the network structure provides performance in cross-validation and determining whether it has a large impact on future predictions. More specifically, we ask which connections in the networks are necessary and which connections are sufficient to generate function prediction performance.

The metric we use for assessment is based on precision-recall curves, using the “average precision” (AP). AP is closely related to the area under the precision-recall curve and is defined as:
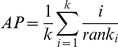
where the gene group (e.g. genes having a certain GO term) contains *k* genes and the algorithm provides a ranking of all genes. Methods performing well will rank genes having the function highly, yielding high average precisions. AP values can then be averaged across groups (e.g. GO terms) to provide a global mean, or MAP for “mean average precision”. The AP values can also be calibrated by comparing them to the distribution of APs obtained for randomly-generated rankings.

**Figure 1:**
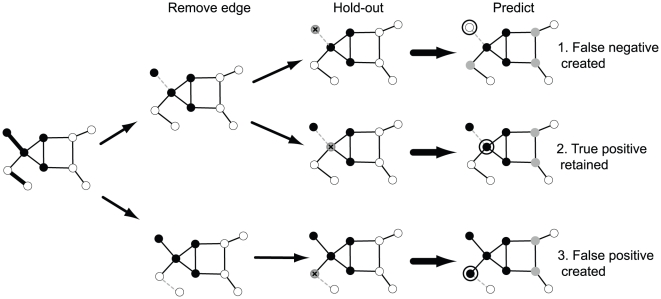
A toy example illustrating how guilt by association can depend on critical edges. At the far left, the input network is shown with the genes having the function (F) we wish to predict shaded black and edges which turn out to be critical are bolded. In the second column, an edge is removed (for simplicity this is only shown for the critical edges). The third column shows three cases of treating a gene as having unknown function (crossed-out grey nodes). At right, the predictions made using neighbor voting are shown (with grey meaning a split decision). In Case 1, a correct prediction depends on one edge; removal of this edge will result in a false negative (circled). In Case 2, there is no single edge that can be removed to cause an error, and the held out gene is correctly predicted. In Case 3, the critical edge of interest is between two genes that lack function F. If this edge is removed, the circled gene is strongly predicted to have function F. In a cross-validation setting, this is considered a false positive. Our experiments show that such effects account for most of the apparent performance of GBA in practice.

In order to characterize the functionality of edges in a network, we use some specific terminology. First, a “functionally relevant edge” is a network edge that connects two genes that share a function. Such edges encode functional information by the GBA principle, but which edges are truly functionally relevant in the network can only be evaluated using known information (or independent verification). Ideally, the network would only contain functionally relevant edges, but this is far from reality; the relevance of an edge may be function-dependent (that is, relevant to some functions and not others) and the networks likely contain edges that are in some sense artifactual. Second, a “critical edge” is one which encodes most of the information about a function that is present in the network (see Figure 1). Criticality can be quantified by the effect removing an edge has on prediction performance (throughout this paper, the term “prediction performance” refers to gene function prediction assessed using cross-validation). Criticality can be used as a proxy for functional relevance, but it must be borne in mind that the relationship is not necessarily straightforward. Finally, an “exceptional edge” is a critical edge for many functional categories; that is, removing an exceptional edge removes functional information for many groups. Exceptionality can be quantified by the fraction of groups which show (for example) a 10% drop in performance when the edge is removed. We use these definitions and quantification approaches throughout this paper. We concern ourselves with questions such as the number and distribution of critical edges and exceptional edges, and finally with the relationship these have to functionally relevant edges.

While we focus on GO terms as the definition of gene function, our findings are not specific to GO (see Text S1, section 2). Indeed this is expected because function based on GO is highly correlated with other gene organization schemes [35]. Our results are also not dependent on the choice of learning algorithm or evaluation metric (see Text S1, section 2).

### Multifunctional connections in the mouse gene network

A key phenomenon is what happens when two highly multifunctional genes are connected in the network. Such edges will tend to be both critical and exceptional. An edge between two genes that share a GO term is useful for prediction of that GO term during cross-validation, thus such edges have an increased probability of being critical compared to randomly selected edges.

**Figure 2:**
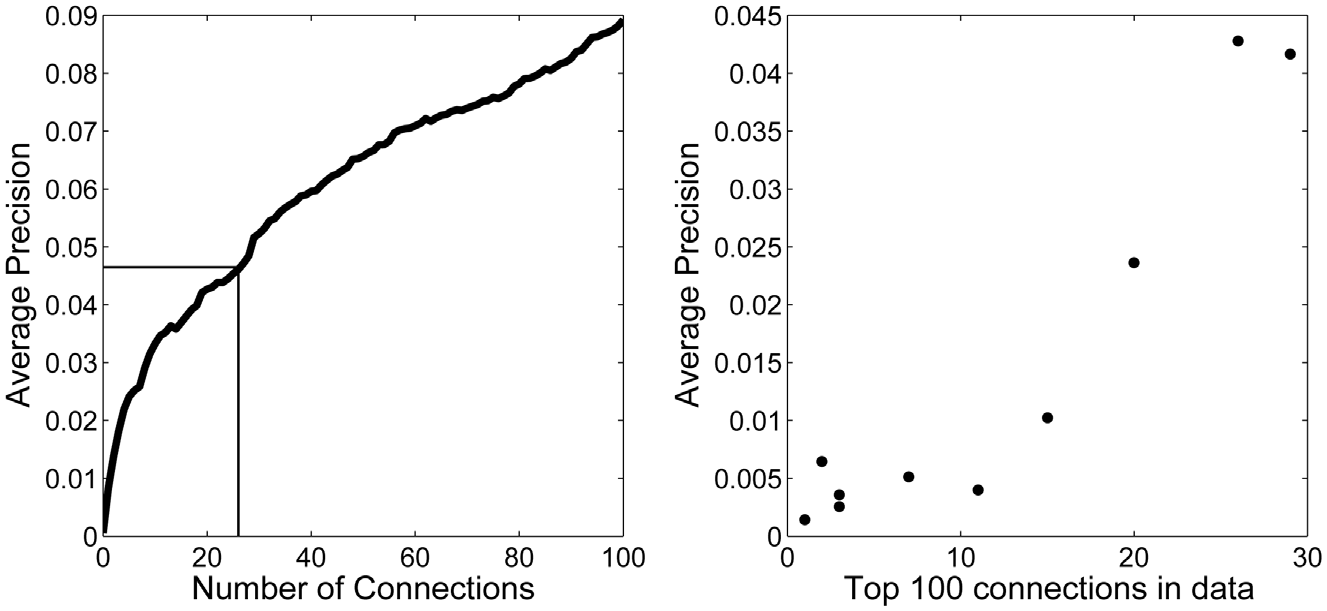
A small number of edges dominate precision-recall in the mouse gene network. A) Average precision as exceptional edges are added, B) Network performance is predicted by overlap with a network of the 100 edges predicted to be most exceptional. The 10 constituent networks of the combined kernel are assessed individually for their precisions and overlap with the 100 edge network.

Intuitively, the more GO terms two connected genes share, the more GO terms for which that edge is likely to be critical. In principle this can have dramatic effects. For example, considering the ∼20000 genes in the mouse genome, a network constructed with just 100 edges among pairs of genes which share the largest number of GO terms yields an MAP across GO terms of ∼0.09, much higher than the expected value of 0.002 if edges were selected at random. That is, the average rank of genes predicted to possess a given function based on their neighbours in the network is substantially elevated across many functions, even using data for only a few genes. This level of performance, with interactions present for only 181 genes, is higher than that obtained with a real network; for a carefully characterized mouse gene network of 4.5 million edges [25], the performance of the real network can be matched with a network of only 23 edges among 45 genes (MAP = 0.047; Figure 2A). These connections are therefore sufficient to generate the results obtained with the real network. Not all of these “most exceptional edges” necessarily exist in a real network, but it turns out that many do and have a dramatic impact on prediction. We assessed 10 mouse gene networks of different types for their degree of overlap with the 100 exceptional edges. The amount of overlapping is strongly predictive of the MAP performance of the real networks (correlation 0.94, Figure 2B). Because these networks incorporate data of diverse types (see Table 1), this suggests the effects of exceptionality are not an artifact of a particular type of network data. In the aggregated mouse network mentioned earlier, removing the 26 edges (0.004% of the total) overlapping with the top 100 exceptional edges from the highest performing network results in a large drop in the MAP (15%). This suggests that a tiny number of edges may account for a large fraction of performance across most GO groups while using no information about most genes and that not only are these connections sufficient to obtain function prediction performance, but they may also be necessary. Because the value of additional edges in the “exceptional edge” network does not dramatically decline when adding more edges (at 150 edges, the MAP is 0.11, far above that of the original network), it is possible a small number of edges accounts for virtually all performance in the real network. These results strongly suggest that in the mouse network, information on gene function is concentrated on too few genes to be of much practical use, at least with regards to how gene function is typically defined (e.g., GO).

**Table 1:** Data sources used for gene function prediction and network construction.

Data type	Data source	Interaction density
Yeast aggregated interactions	MPACT [2], DIP [4], MINT [1], BioGRID [41], Fields [42], Costanzo et al [40]	0.38%
Optimized yeast interactions	Yeastnet [23]	0.51%
Yeast genetic interactions	Costanzo et al [40]	0.22%
Human aggregated protein interactions	iRefIndex [48], InnateDB [49], HPRD [50], BIND [51], OPHID [52], MINT [53]	0.047%
Mouse expression profiles	Mouse gene atlas [25], [57]	0.31%
	Zhang et al [25], [58]	0.26%
	SAGE mouse atlas [25], [59]	0.30%
Mouse sequence	Pfam [25], [60]	0.27%
	InterPro [25], [61]	0.28%
Mouse protein interaction	OPHID [52]	0.12%
Mouse phenotypes	MGD [25], [62]	0.06%
Mouse conservation profile	EnsMart [25], [63]	0.29%
	Inparanoid [25], [64]	0.28%
Mouse disease associations	OMIM [25], [65], NCBI [65], [66]	0.0013%
Mouse aggregated data	Mousefunc [25]	1.9%

Primary networks assessed individually and in aggregate are shown with sparsities calculated over the full genes set.

### Yeast gene network exceptional edges

We performed a detailed analysis of multiple *Saccharomyces cerevisiae* gene interaction networks [1], [2], [4], [40], [41], [42], which are more tractable to analyze exhaustively than the mouse networks due to their smaller size (much sparser as well as having 1/3 the number of genes). We propose that these networks (and their aggregate) are representative of the highest-quality data available for gene function analysis.

**Figure 3:**
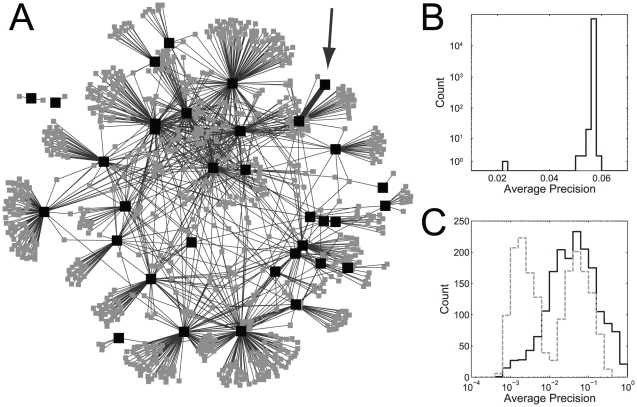
Critical edges exist in networks. A) The subnetwork for a GO group (“Cellular polysaccharide biosynthetic process”) is shown with in-group connections shaded in black and outgroup connections in grey. The arrow points to a critical connection. B) The distribution of average precisions resultant from the family of network differing by removing one connection from the original full network. One connection has a huge effect. C) Including only critical edges (grey dashed) results in performance that is similar to the original network (solid black), in part, or almost completely absent.

Using an aggregate of five of the networks, we identified critical edges by removing single edges and testing the average precision of each of 1746 GO terms (see Methods), for each edge in the network. This yielded a dataset consisting of gene function prediction performance for each GO term in each of 72481 networks, each differing from the complete network by just one edge. This data set allows us to determine which individual connections are necessary to generate meaningful predictions for any given function; it can be visualized as a matrix of 72481 connections by 1746 average precisions of gene function prediction for that GO group using that network (missing one connection). A critical edge, then, is one in which edge removal changes precision substantially for a given GO group, while exceptionality can be determined by aggregating the criticality of a connection across all GO groups. Removing any single edge usually has little effect on performance for any given GO term, but when it does have an effect, it is drastic. In Figure 3A, a sub-network for a representative GO term is shown; the distribution of the average precision values for this GO term with edges removed contains an extreme outlier (Figure 3B). These genes have 27 unique interactions with one another and over 1200 connections to other genes. The average precision of this group using the complete network is 0.057 (p<10^−4^), high enough to be of practical importance to an experimentalist (a functionally related gene is expected among the top 20 genes associated with genes within the GO group). However, the majority of functional information comes from a single edge, in which a gene within the GO group has a lone connection to another gene within the GO group. From the point of view of function prediction, this is problematic since most predictions going forward may have nothing to do with that edge or the two genes the edge links, and thus lack any evidence for being correct.

Using this edge removal method, for each of 1746 GO terms, we identified the most critical edge. A single edge contributes very strongly to performance for the majority of GO terms, with an average contribution of 39% (see Figure S1). This means that when predictions were made in cross-validation, at least one of the folds had a ranking in which a true positive “hit” gene ranked highly due to one connection. This includes many GO groups where removing an edge has an effect greater than 100% (removing the edge dropped performance below that expected on average by chance; fixing the maximum possible effect at 100% yields an average effect of 24%). We obtained very similar results to these when testing six networks individually (our five constituent networks plus YeastNet [23]), with two informative exceptions that had fewer GO groups with a critical edge (see Figure S2). In the case of YeastNet this is because the network had been specifically tuned to reinforce GO learning in that edges were added or removed using knowledge from GO [23]. In contrast, the yeast genetic interaction network [34] suffers from a very low number of significantly learnable GO groups (only 3% of GO group have average precisions more than 0.01 above the expected value, in contrast to the BioGRID protein interaction network [41], where 67% of GO groups have at least that level of performance); networks without learnable information also don't have critical information (an alternative representation of genetic interactions, which does show critical edges concomitant with higher performance, is considered in Text S1, section 3).

**Figure 4:**
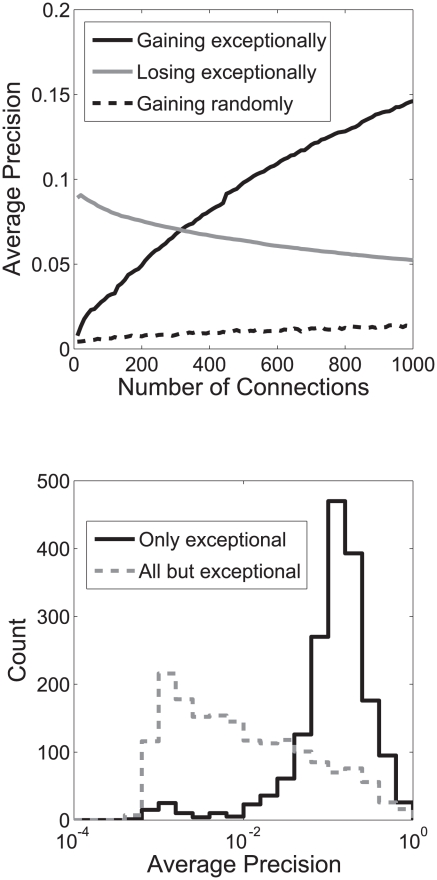
Functional information is not distributed throughout the network. A) Removing exceptional edges from the network causes a decline in performance, while adding them to an empty network causes a very rapid rise in performance, above even that possessed by the full network. B) Removing all of the 4870 potentially exceptional edges from the network removes most of its performance (black solid line), while adding only those edges (grey dashed) yields high performance across all GO groups.

It turns out that many of the GO groups share the same “most critical edge” (see Figure S3): we identified 100 edges in the aggregate yeast network that are the most critical for ∼1/3 of the GO groups. Using just these edges for prediction of all GO terms we would expect a bimodal distribution of performance, in which the ∼1/3 of the GO groups for which the 100 edges are critical would have average precisions of approximately 60% of the full matrix (since critical edges account for ∼40% of performance on average), while 2/3 of GO groups would have a performance drawn from the null distribution with most average precisions below 0.005. In fact, as shown in Figure 3C, more GO groups are learnable than expected (1/2), due to the presence of “nearly critical” edges (see Text S1, section 4). Adding edges by their average degree of criticality across all GO groups (their exceptionality), we see the network performance quickly improves above that of the full network (Figure 4A).

If we define a critical edge as one affecting the learnability of at least one GO group by 10%, we obtain a network of 4870 edges from the yeast data. We consider this larger set of edges to determine which interactions may be necessary (rather than merely sufficient) to generate function prediction performance. While a very small number of edges are sufficient, it is possible that redundancy in the network makes removing those few edges insufficient to remove all functional information. Interestingly, these 4870 edges are not necessarily between two members of the GO group for which the edge is critical (an “internal” edge) and in 50% of these GO groups, at least one of the connections was an external critical connection. Sometimes an edge is critical because it correctly documents non-membership (an “external” edge). In this case, a non-member gene connected to an in-set gene would be highly ranked were it not for a critical connection to a gene outside the set. The earlier ranking of connections by their exceptionality gives a better sense of what connectivity is sufficient to generate gene function prediction performance. A network with as few as 350 connections generates better function prediction performance in the remainder of the 72131 connections. As in the mouse network, these critical connections provide essentially all of the learnable information in the network (Figure 4B). These edges are also important even in the context of the full network, since their removal causes a significant decline in performance (Figure 4B), and while their removal does not remove all functional information from the network, they are also not redundant with it (as seen in the decline in precision-recalls)

We noted that there is a small subset of GO groups with very high learnability in the full network data (average precision>0.5). No groups have such high performance when only exceptional edges are used, suggesting something other than critical edges is responsible. A cursory inspection reveals these outliers are highly enriched for GO terms representing protein complexes. Such GO terms have an extremely high MAP on average (0.33; N = 91; Figure S4; Text S1, section 5). The network properties of these groups are also unusual, with a “clique-like” structure in contrast to other GO terms that tend to have very sparse connections among the members (Figure S4). Because of this property, we would not expect any edge to be critical. In addition, edges within the complex have a very different “meaning” than edges connecting complex members with genes outside. In particular, the former can be used to infer complex membership, but the latter obviously cannot. There is no reason to think the high learnability of protein complexes would reflect well on predicting the function of genes interacting with but not in the complex; nor can it be used to infer anything about the learnability of other functional groups.

A remaining issue is whether there are *any* GO terms for which we might expect some generalizable predictability. For this to be the case, the group should be learnable in cross-validation, but not have any especially – meaning dominantly - critical edges (or equivalently have many edges strongly improving average precision). This would at least increase the confidence that other edges (used for extracting novel information) are functionally relevant. Unfortunately GO groups that lack critical edges altogether tend not to be learnable in cross-validation and very rarely do GO groups have very many critical connections (Table S1).

### Pruning the network for functional links

We argue that the presence of exceptional edges is a problem, and ideally the network would not contain them. This is because they concentrate most of the apparent functional information in a tiny fraction of the network and are not specific to any one function, and therefore cannot provide specific functional information about most genes. On the other hand, critical edges are the only readily available correlate for functionally relevant connections. Thus the ideal network would contain only critical edges (which are hopefully the functionally relevant ones), but few exceptional edges. However, it is not satisfactory to evaluate criticality using impact on learnability, as this would result in overfitting. It is therefore desirable to identify more general properties of critical edges other than their impact on learnability. We sought a correlate of criticality which can be used to prioritize some connections over others.

Based on our previous research showing that high node degree genes are generic in their functionality [35], we suspected that edges involving genes with high node degree (hubs) are less likely to be critical. This is because losing a gene's only connection is more likely to damage learning performance than removing one of dozens. In addition, hubs may represent highly-studied genes potentially more open to the accumulation of false positive connections. In Figure S5, we can see that the fraction of critical edges a gene possesses decreases as a function of its total number of connections. We propose, then, to prune the network by privileging connections on low node degree genes. This is consistent with our previous work showing that hubs tend to attract computational predictions at the expense of less-well-characterized genes (“rich get richer”) [35].

**Figure 5:**
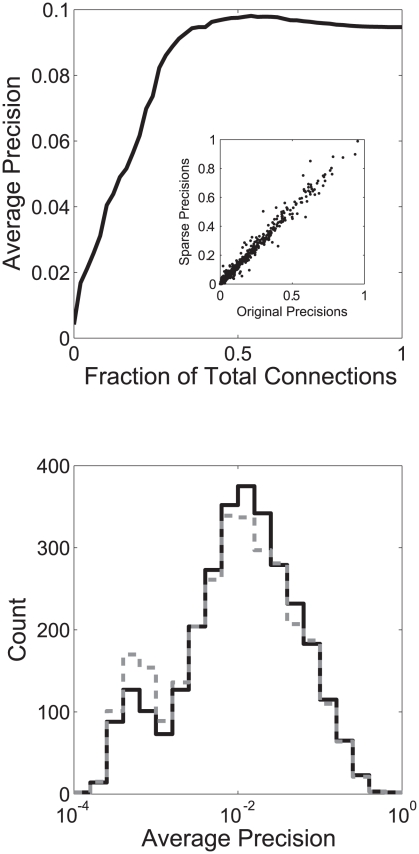
Critical edges are identifiable from network structure. A) Performance over networks as connections are added to an empty network based on node degree (low node degree connections get added back first). Performance rises to the same as the real network well before the network is fully reconstructed. B) The sparser human network (grey) shows a distribution of GO performances similar to the original network (black); slightly higher in most GO groups, with slightly lower coverage.

This pruning yields a network that, even with 1/2 of connections removed, performs similarly to the original network (Figure 5A). The specific predictions made are also very similar, with genes that are predicted strongly in the original network tending to have similar relative ranks in the pruned network (Text S1, section 6 and Figure S7). While this has not necessarily improved the situation with respect to generalizing, removing edges from the network implies that fewer predictions will be made in the first place, which is helpful in that it removes potentially misleading results. It further suggests that, at least with respect to GO, gene networks contain many irrelevant edges that can potentially be identified using principled means. We tested this pruning procedure in an independently constructed network of human protein interaction data. We find that pruning the human network by half did not remove functional information, as determined from the function predictions (Figure 5B). We confirmed that this network pruning worked by preferentially selecting exceptional edges by examining the human network for criticality, as in the yeast network. We found roughly comparable criticality, with the 1475 GO groups with average precision above 0.01 having a critical connection average effect of 44% of their performance (the threshold of 0.01 allows for the fact that fewer GO groups are learnable from the human data). One possibility is that the ability to discern criticality in both networks merely reflects interactions present in both networks through homologies. In fact, mapping the criticality of connections between the two networks through homology reveals no correlation between the two (r = −0.02); what is critical in one network is no more likely than average to be critical in the other.

### Functional connectivity and network structure

We have suggested that a major problem with the existence of exceptional edges is that they reduce supposedly “network-wide” properties to the properties of a very small part of the network. As a specific example of this problem (beyond describing the information encoded in networks), we consider a well-studied network property, whether the network is scale free (or at least scale-free-like, with a very heavy tail to the degree distribution) [43]. Our original yeast protein interaction network has a “scale free” structure, as exhibited in the distribution of its node degree (see Figure S6). However, our results show that connections of high node degree genes are preferentially free of specific functional information, suggesting that the two most famous properties of biological networks, functional association and approximate scale freeness, are largely independent. To demonstrate this, we perform the pruning by node degree in the yeast network which we know improves GBA performance, but has the effect of truncating the node degree distribution (Figure S6). While truncated power-law distributions for networks have been previously discussed [44], this degree of scaling is generally not reported, and there is clearly a dominant scale in the network. The pruned network node degree distribution is well characterized by its average node degree of 12 and the distribution does not appear at all to follow a power law distribution. The power law node degree structure in this network was preferentially encoded in connections that contain no known functional information.

### Characterizing exceptional edges

Because exceptional edges preferentially encode function, one reasonable expectation might be that they are higher quality in terms of their experimental support. To test this, we employed the HIPPIE database (http://cbdm.mdc-berlin.de/tools/hippie/) which characterizes protein interactions by the strength of evidence supporting them (including experimental techniques employed). There is a weak but significant rank correlation between exceptionality and data quality as judged by HIPPIE (r = 0.09, p<0.01); higher quality data is more likely to encode exceptionality. While we would not expect a particularly strong trend across the network at large (due to our emphasis on the role of outliers), another factor is serving to weaken the correlation. Edges that encode no known function, and therefore accrue exceptionality only by virtue of encoding non-membership in a function (these are the “external” edges discussed above), show a trend in the opposite direction to those edges which largely encode functionality “internally” (or are strongly functionally relevant as judged by a high semantic similarity of GO annotations; Jaccard index>0.75). Edges which encode non-functionality are significantly associated with better quality linkage (p<0.05), while those that encode direct functionality are significantly associated with lower quality linkage (p<0.05). One possible interpretation of this result is that it reflects differences in the degree to which genes are studied, and that highly multi-functional genes may more readily accumulate “high quality” interaction data with one another than they may accumulate low-quality connections with less studied genes [45].

To further examine how exceptional edges arise, we looked at the role they play in randomly constructed networks, in which any given connection is equally likely to occur. We first conducted experiments using randomly defined “GO groups” of fixed size (20 genes; see Methods). The distribution of MAP values across 1000 random networks was approximately normal (p∼0.5, Kolmogorov-Smirnov test), but as expected most networks generated in this way do not yield significantly high MAP values. We used the statistical parameters from our initial simulations to pick a MAP threshold (more than 3 standard deviations from the mean) for 100000 random networks. Averaging across the 876 such networks produced during our simulation, we obtain exceptional edges in the sense that the 24 connections most frequently reoccurring across those networks yields a (very small) network which performs well (z-score>3; that is, above the threshold used to select the 876 individual networks). Examining these edges, they have an elevated semantic similarity in their “pseudo-GO” annotations (Jaccard similarity of 0.09 compared to an expected value of 0.01; p<0.01). Based on this, it appears that exceptional connections occur in high-scoring random networks for the simple reason that it is easier to accidentally obtain small number of highly impactful (exceptional) edges than many edges with smaller effects on performance (the latter would be expected if there was systemic encoding of function throughout the network). We obtained similar results with the same type of random networks trained using on the real Gene Ontology, suggesting that the appearance of criticality in gene function prediction is not an artifact of GO structure.

## Discussion

Gene function is commonly thought of as being a network property, and in the types of networks considered here, it is often assumed that gene function is “encoded” in the associations. Our results challenge this assumption, since the primary evidence for the distribution of function in the networks are things like patterns of GO annotations. We have demonstrated that in a wide variety of gene networks, known information on gene function is concentrated in a handful of “exceptional edges”. One implication is that it is very misleading to use functional analysis such as GBA to bolster the case that a gene network is of high quality. A second implication is that current computational strategies for predicting gene function from networks are deeply flawed. We also provide evidence that the “scale-free-like” behaviour of gene networks is independent of gene functional relationships, raising the question of how such properties should be interpreted.

### Scalability of GBA

One way of viewing our findings is that the GBA principle, which is fruitfully applied by biologists on a small scale when analyzing genes one at a time, does not scale easily to networks. Our results suggest that, for any given function, most associations are either useless or misleading. This is likely to be partly due to noise but also the fact that large networks are not constructed with a particular gene (or function) in mind. Small-scale studies do not escape this problem, but when testing the associations of a single gene under more controlled conditions, especially in “function-specific” conditions, biologists can more efficiently reject spurious findings and enrich for functionally-relevant associations. For these reasons we suspect that large-scale attempts to analyze gene function will continue to be frustrated by the mismatch between the content of the network and “gene function” as it is currently systematized. The notable exception is protein complexes. The problem with the mismatch between gene function and the networks could also be seen as lying either with GO (and other systems of defining gene function), or with the networks themselves. Indeed, our results suggest that the apparent agreement of GO and gene networks is largely an illusion (again, with the exception of protein complexes). Thus function information might be extracted from networks, but not routinely using schemes like GO as a guide. However, as mentioned above it is also likely that the gene networks themselves are problematic, in that they likely contain many edges that are not functionally relevant. The “ever more data” approach common to the field runs the risk of filling gene networks with false positives as the occasional errors in individual experiments are aggregated, and it is very difficult to prove the lack of an interaction. In support of this, protein interactions in the BioGRID network have declined in average apparent functionality over the past fifteen years (Figure S8), with the Jaccard similarity for connections added in a given year declining on average (r = −0.95, p<0.01). This problem is exacerbated by the necessary reliance on computation, which makes it harder to see which part of the data is providing learning performance.

### Reinterpreting networks

It seems one has to decide whether it makes more sense to “fix” the networks so that they are more functionally relevant, or to discard GO and its relatives for this purpose in favour of an alternative (potentially equally problematic) that matches the networks better. The former makes sense if one is interested in predicting GO group membership. While this is treated as an important goal by many, it has in fact been thrust upon the field as a default; predicting GO terms has become a proxy for predicting gene function in general. Our results on network pruning by node degree suggest that current networks can be cleaned up extensively without hurting GO prediction in cross-validation, but generalizing to make useful new predictions is still a very serious problem. Replacing GO also seems very challenging: all current systematizations of gene function that we are aware of are currently highly correlated with GO (or indeed directly mapped to GO), such as KEGG, MIPS, EC numbers, Pfam, and so on; we are certainly not aware of any systematization which is more learnable than GO (if there was, GO would not be used as much for this purpose).

There is at least a third alternative, to use the network itself to define function, where the main function to be “predicted” is “gene X interacts with gene Y”. This is of course a common exploratory way to use the data (“What is my gene connected to?”), but the quality of the network itself becomes paramount, and as a definition of function it verges on the trivial. Furthermore, “gene X interacts with gene Y” is most definitely not a function that is any meaningful sense “distributed” in the network. Guilt by association (in the most general sense) has provided essentially the sole principled interpretation of network data from a functional perspective. Without it, rather than providing information on function, connectivity in this sense is only information on mechanisms; we must essentially switch from a top-down perspective, informed by GBA, to a bottom-up perspective based on the specific insight interactions provide. If interaction data has a purely observational meaning, then network quality can only be assessed by its replicability and consistency, standards by which most network data would probably perform poorly. Other network-derived definitions of gene function such as “hubbiness” or “betweenness centrality” [46] that are less sensitive to network quality are potentially more useful, but only help throw the limitations of the network for deriving more precise statements about gene function into relief. We note that while we have not directly addressed all variants of GBA which focus on predicting protein interactions, regulatory relationships, or the effects of mutations, these either amount to making statements about the network itself (filling in missing edges, or interpreting an edge), or are likely to behave similarly to GO prediction. We conclude that gene networks encode information on gene function, but primarily in ways that are highly localized and with very limited predictive ability.

### How should networks encode function?

Many gene function prediction methods explicitly treat “protein-complex”-like structures (cliques) as an optimal way to encode function (e.g. [25], [47]). Functional information encoded in this way is readily retrievable by algorithmic means and shows optimal “guilt by association”. While this captures some functions, it is not what one would expect or desire as a general property of a gene network for function prediction purposes. If those cliques are not connected together (allowing perfect GBA for the functions encoded by the clique), one cannot predict any additional functions. On the other hand, if the cliques are connected together, one must ask what the desired structure of that “coarser” network should be (treating cliques like genes). If the answer is that it should also be clique-like in order to optimize GBA, one rapidly exhausts the network in a small set of hierarchical modules. This might be satisfactory if one supposes that gene function is strongly hierarchical (and also fairly simply organized), but this is clearly counter to the state of affairs. Indeed, in real networks genes with similar functional annotations tend to be connected together not just for “protein complexes” but for most functions (the GO annotation Jaccard similarity matrix in our yeast data yields a high MAP of 0.65).Thus, it is possible in principle to encode functionality more broadly, without requiring cliques, and without relying on multiple networks to obtain specificity. While we have highlighted the role of exceptional edges as a problem, we also believe that recognizing the importance of exceptional edges more clearly replicates the way biologists work with data; thus, the classification of interactions with greater detail is a step toward “fixing” guilt by association.

### Conclusions

Our results lead to some concrete recommendations for gene network analysis. First, if one is assessing network quality using GBA-like approaches, it is essential to test the effect of critical edges. Because exhaustively identifying critical edges is computationally intensive, our approach for pruning edges based on node degree provides a useful and easy-to-compute diagnostic. If pruning (say) ½ of the network has little effect on GBA performance, it is obvious that most of the (measurable) functionally-relevant information is concentrated in a very small fraction of the network, making global statements about network quality unlikely to be of use. A separate assessment of the network for the completeness of recovery of protein complexes is also reasonable, bearing in mind that these have very distinct properties. Our second set of recommendations is directed at investigators who are attempting to create gene function prediction tools. Cross-validation performance will be a useless measure of the quality of new predictions unless it is first shown that, for any given classification task, performance is not due to a single edge. Again, doing this exhaustively is computationally expensive, but our results provide some rules of thumb. One should test the effect of the removal of edges that involve an in-group gene; such edges are at least enriched for critical edges (bear in mind that a critical edge can involve two out-of-group genes, so negative results for this test are not conclusive). These tests should be used in conjunction with our previous suggestion that learning performance be compared to that provided by node-degree ranking [35].

## Methods

Additional information on the methods, implementations and data is available at www.chibi.ubc.ca/critcon.

Gene networks: The mouse network data consisted of 10 data matrices representing associations among 21603 genes, with 774 GO groups (10–300 genes each) being used for assessment as in [25]. Our yeast PPIN was obtained by aggregating data from [1], [2], [4], [40], [41], [42] and contained 72481 unique interactions Our human PPIN was obtained by aggregating data from [48]–[53] and contained 100623 unique interactions. Additional detail on the component networks is provided in Table 1.

Gene lists: We analyzed the list of 20710 human genes from the UCSC GoldenPath database [54] “known gene” table. The 6200 yeast gene list was obtained from NCBI [55]. The mouse gene list was as used in [25].

Algorithm: For guilt by association analysis, we ranked genes by a voting scheme within the training set (by ranked coexpression) relative to genes outside the training. Despite its simplicity, this method gives performance comparable to the best-performing algorithms [56], with the benefit of being extremely fast.

Cross-validation: Eight-fold cross-validation was used in assessing the mouse data, and three-fold cross validation was used to detect critical connections in the yeast and human data and for assessment consistency. Performance was assessed by taking the precision averaged across all true positives within a particular testing set (that is, the discrete sum), yielding the area under the precision-recall curve or average precision (see Text S1, section 1). Our findings hold for other measures such as receiver operating characteristic (ROC) curves, but as shown in [35], ROC curves are sensitive to node degree effects. In contrast precision-recall curves allow us to more effectively isolate the effect of critical edges.

Critical edges were detected by performing the full gene function cross-validation across all GO groups for each of the networks resultant from removing one edge from the full network, in both the human, yeast, and constituent networks. Exceptional edges were chosen by aggregating the average precision the network resultant from a given edge being removed, across all GO groups. The more performance is degraded across all GO groups, the higher the exceptionality of the edge. Exceptional edges were predicted by selecting the gene pair possessing the largest number of overlapping GO functions, weighting each GO function by the inverse of the number of times it had already been used to add gene pairs, and repeating until the desired number of edges were obtained.

Simulations: Random networks were constructed of size 1000 genes with sparsity 0.002 (1000 edges) and assessed for functional performance using a random set of gene groupings (100 groups of size 20). MAP across the groups was assessed using neighbour-voting, and those networks scoring more than three standard deviations above the mean of 1000 simulations were aggregated to determine commonalities in their connectivity.

## Supporting Information

Figure S1 Figure showing most GO groups are strongly affected by removing a single connection. Shown is the fraction of average precision performance contributed by a single critical edge for each GO group.
(EPS)

Figure S2 Figure showing most GO groups in most networks are affected by removing a single connection. For each of our constituent networks, plus the Yeastnet network, we assess the importance of removing each connection in the context of that network. Yeastnet and the genetic interaction network are somewhat outliers due to optimization with respect to GO (Yeastnet) and low performance of direct interactions in the genetic interaction network.
(EPS)

Figure S3 Figure showing most GO terms share critical edges. The number of GO groups with their critical edges included rises more rapidly than the number of connections, due to overlap of critical edges; we call such critical edges “exceptional”.
(EPS)

Figure S4 Figure showing protein complexes have distinctive properties. A) Protein complexes have exceptionally high precision-recalls in GBA B) The density of in-group connections is very high in protein complexes, and uniquely so, so that if a given group (by GO) of genes forms a fully connected sub network, it is assuredly a protein complex. C) Because of their density of in-group connections, protein complexes contribute very strongly to the GO groups not dominated by critical edges, despite their low prevalence.
(EPS)

Figure S5 Figure showing that in the yeast network, node degree is a correlate of average criticality of the connections for that gene. For each node degree, the fraction of connections which are critical for that node are shown, and clearly declines with increasing node degree.
(EPS)

Figure S6 Figure showing heavy-tails are characteristic of the original protein interaction network but not the pruned network. A) The node degree distribution of the original network is shown, as well as the power-law fit, showing the very heavy tail. B) The node-degree distribution of the pruned network is shown, as well as the power-law fit, showing no heavy tail, as well as exhibiting a characteristic node degree to the distribution.
(EPS)

Figure S7 Figure showing pruning retains significant predictions. A) For a particular GO group, the average precision of individual genes from complete prediction is shown along the X axis, and the similarity of those precisions to those determined in the pruned network is shown along the y-axis, by rank. B) The trend between precisions pre and post pruning is shown averaged across all GO groups, with the black line individuating the mean for a given decile, and the grey lines showing the standard deviation.
(EPS)

Figure S8 Figure showing interaction reports are becoming less functional over time. The relationship between the year of an interaction report (by citation in BioGRID), and the average functionality (as encoded by semantic similarity) of edges is plotted.
(EPS)

Table S1 Table showing unusually critical gene ontology groups. All GO groups with 10% or more of their connections exhibiting more than a 0.01 effect upon average precision. The vast majority of GO groups have relatively few connections that might be thought to encode functional information about the group.
(DOC)

Text S1 Text describing additional experiments and results, including details on cross-validation (section 1), alternatives to “basic GBA” and critical edges (section 2), genetic interaction profile data (section 3), additivity of critical edges effects (section 4), protein complexes (section 5), and pruning networks (section 6).
(DOC)

## References

[1] Cesareni G, Chatr-aryamontri A, Licata L, Ceol A (2008). Searching the MINT database for protein interaction information.. Curr Protoc Bioinformatics Chapter.

[2] Guldener U, Munsterkotter M, Oesterheld M, Pagel P, Ruepp A (2006). MPact: the MIPS protein interaction resource on yeast.. Nucleic Acids Res.

[3] von Mering C, Krause R, Snel B, Cornell M, Oliver SG (2002). Comparative assessment of large-scale data sets of protein-protein interactions.. Nature.

[4] Xenarios I, Salwinski L, Duan XJ, Higney P, Kim SM (2002). DIP, the Database of Interacting Proteins: a research tool for studying cellular networks of protein interactions.. Nucleic Acids Res.

[5] Horan K, Jang C, Bailey-Serres J, Mittler R, Shelton C (2008). Annotating genes of known and unknown function by large-scale coexpression analysis.. Plant Physiol.

[6] Lee HK, Hsu AK, Sajdak J, Qin J, Pavlidis P (2004). Coexpression analysis of human genes across many microarray data sets.. Genome Res.

[7] Saito K, Hirai MY, Yonekura-Sakakibara K (2008). Decoding genes with coexpression networks and metabolomics – ‘majority report by precogs’.. Trends Plant Sci.

[8] Pellegrini M, Marcotte EM, Thompson MJ, Eisenberg D, Yeates TO (1999). Assigning protein functions by comparative genome analysis: protein phylogenetic profiles.. Proc Natl Acad Sci U S A.

[9] Pu S, Ronen K, Vlasblom J, Greenblatt J, Wodak SJ (2008). Local coherence in genetic interaction patterns reveals prevalent functional versatility.. Bioinformatics.

[10] Tong AH, Lesage G, Bader GD, Ding H, Xu H (2004). Global mapping of the yeast genetic interaction network.. Science.

[11] Typas A, Nichols RJ, Siegele DA, Shales M, Collins SR (2008). High-throughput, quantitative analyses of genetic interactions in E. coli.. Nat Methods.

[12] Simonis N, Rual JF, Carvunis AR, Tasan M, Lemmens I (2009). Empirically controlled mapping of the Caenorhabditis elegans protein-protein interactome network.. Nat Methods.

[13] Hibbs MA, Hess DC, Myers CL, Huttenhower C, Li K (2007). Exploring the functional landscape of gene expression: directed search of large microarray compendia.. Bioinformatics.

[14] Kaplan S, Bren A, Dekel E, Alon U (2008). The incoherent feed-forward loop can generate non-monotonic input functions for genes.. Mol Syst Biol.

[15] Balazsi G, Barabasi AL, Oltvai ZN (2005). Topological units of environmental signal processing in the transcriptional regulatory network of Escherichia coli.. Proc Natl Acad Sci U S A.

[16] Aerts S, Lambrechts D, Maity S, Van Loo P, Coessens B (2006). Gene prioritization through genomic data fusion.. Nat Biotechnol.

[17] Hess DC, Myers CL, Huttenhower C, Hibbs MA, Hayes AP (2009). Computationally driven, quantitative experiments discover genes required for mitochondrial biogenesis.. PLoS Genet.

[18] Ashburner M, Ball CA, Blake JA, Botstein D, Butler H (2000). Gene ontology: tool for the unification of biology. The Gene Ontology Consortium.. Nat Genet.

[19] Oliver S (2000). Guilt-by-association goes global.. Nature.

[20] Mani R, St Onge RP, Hartman JLt, Giaever G, Roth FP (2008). Defining genetic interaction.. Proc Natl Acad Sci U S A.

[21] Arabidopsis Interactome Mapping Consortium (2011). Evidence for network evolution in an Arabidopsis interactome map.. Science.

[22] Mukhtar MS, Carvunis AR, Dreze M, Epple P, Steinbrenner J (2011). Independently evolved virulence effectors converge onto hubs in a plant immune system network.. Science.

[23] Lee I, Li Z, Marcotte EM (2007). An improved, bias-reduced probabilistic functional gene network of baker's yeast, Saccharomyces cerevisiae.. PLoS One.

[24] Myers CL, Robson D, Wible A, Hibbs MA, Chiriac C (2005). Discovery of biological networks from diverse functional genomic data.. Genome Biol.

[25] Pena-Castillo L, Tasan M, Myers CL, Lee H, Joshi T (2008). A critical assessment of Mus musculus gene function prediction using integrated genomic evidence.. Genome Biol.

[26] Hishigaki H, Nakai K, Ono T, Tanigami A, Takagi T (2001). Assessment of prediction accuracy of protein function from protein–protein interaction data.. Yeast.

[27] Tsuda K, Shin H, Scholkopf B (2005). Fast protein classification with multiple networks.. Bioinformatics.

[28] Vazquez A, Flammini A, Maritan A, Vespignani A (2003). Global protein function prediction from protein-protein interaction networks.. Nat Biotechnol.

[29] Wolfe CJ, Kohane IS, Butte AJ (2005). Systematic survey reveals general applicability of “guilt-by-association” within gene coexpression networks.. BMC Bioinformatics.

[30] Zhou X, Kao MC, Wong WH (2002). Transitive functional annotation by shortest-path analysis of gene expression data.. Proc Natl Acad Sci U S A.

[31] Chua HN, Sung WK, Wong L (2006). Exploiting indirect neighbours and topological weight to predict protein function from protein-protein interactions.. Bioinformatics.

[32] Weston J, Elisseeff A, Zhou D, Leslie CS, Noble WS (2004). Protein ranking: from local to global structure in the protein similarity network.. Proc Natl Acad Sci U S A.

[33] Lee I, Ambaru B, Thakkar P, Marcotte EM, Rhee SY (2010). Rational association of genes with traits using a genome-scale gene network for Arabidopsis thaliana.. Nat Biotechnol.

[34] Costanzo M, Baryshnikova A, Bellay J, Kim Y, Spear ED (2010). The genetic landscape of a cell.. Science.

[35] Gillis J, Pavlidis P (2011). The impact of multifunctional genes on “guilt by association” analysis.. PLoS One.

[36] Albert R (2005). Scale-free networks in cell biology.. J Cell Sci.

[37] Gomez SM, Lo SH, Rzhetsky A (2001). Probabilistic prediction of unknown metabolic and signal-transduction networks.. Genetics.

[38] Zhang B, Horvath S (2005). A general framework for weighted gene co-expression network analysis.. Stat Appl Genet Mol Biol.

[39] Tanaka R, Yi TM, Doyle J (2005). Some protein interaction data do not exhibit power law statistics.. FEBS Lett.

[40] Costanzo M, Baryshnikova A, Bellay J, Kim Y, Spear ED (2010). The genetic landscape of a cell.. Science.

[41] Breitkreutz BJ, Stark C, Reguly T, Boucher L, Breitkreutz A (2008). The BioGRID Interaction Database: 2008 update.. Nucleic Acids Res.

[42] Schwikowski B, Uetz P, Fields S (2000). A network of protein-protein interactions in yeast.. Nat Biotechnol.

[43] Barabasi AL, Albert R (1999). Emergence of scaling in random networks.. Science.

[44] Mossa S, Barthelemy M, Eugene Stanley H, Nunes Amaral LA (2002). Truncation of power law behavior in “scale-free” network models due to information filtering.. Phys Rev Lett.

[45] Yu X, Ivanic J, Memisevic V, Wallqvist A, Reifman J (2011). Categorizing biases in high-confidence high-throughput protein-protein interaction data sets.. Mol Cell Proteomics.

[46] Joy MP, Brock A, Ingber DE, Huang S (2005). High-betweenness proteins in the yeast protein interaction network.. J Biomed Biotechnol.

[47] Mostafavi S, Ray D, Warde-Farley D, Grouios C, Morris Q (2008). GeneMANIA: a real-time multiple association network integration algorithm for predicting gene function.. Genome Biol.

[48] Razick S, Magklaras G, Donaldson IM (2008). iRefIndex: a consolidated protein interaction database with provenance.. BMC Bioinformatics.

[49] Lynn DJ, Winsor GL, Chan C, Richard N, Laird MR (2008). InnateDB: facilitating systems-level analyses of the mammalian innate immune response.. Mol Syst Biol.

[50] Prasad TS, Kandasamy K, Pandey A (2009). Human Protein Reference Database and Human Proteinpedia as discovery tools for systems biology.. Methods Mol Biol.

[51] Gilbert D (2005). Biomolecular interaction network database.. Brief Bioinform.

[52] Brown KR, Jurisica I (2005). Online predicted human interaction database.. Bioinformatics.

[53] Ceol A, Chatr Aryamontri A, Licata L, Peluso D, Briganti L (2010). MINT, the molecular interaction database: 2009 update.. Nucleic Acids Res.

[54] Kent WJ, Sugnet CW, Furey TS, Roskin KM, Pringle TH (2002). The human genome browser at UCSC.. Genome Res.

[55] NCBI (2002). The NCBI handbook [Internet].

[56] Gillis J, Pavlidis P (2011). The role of indirect connections in gene networks in predicting function.. Bioinformatics.

[57] Su AI, Wiltshire T, Batalov S, Lapp H, Ching KA (2004). A gene atlas of the mouse and human protein-encoding transcriptomes.. Proc Natl Acad Sci U S A.

[58] Zhang W, Morris QD, Chang R, Shai O, Bakowski MA (2004). The functional landscape of mouse gene expression.. J Biol.

[59] Siddiqui AS, Khattra J, Delaney AD, Zhao Y, Astell C (2005). A mouse atlas of gene expression: large-scale digital gene-expression profiles from precisely defined developing C57BL/6J mouse tissues and cells.. Proc Natl Acad Sci U S A.

[60] Finn RD, Mistry J, Schuster-Bockler B, Griffiths-Jones S, Hollich V (2006). Pfam: clans, web tools and services.. Nucleic Acids Res.

[61] Mulder NJ, Apweiler R, Attwood TK, Bairoch A, Bateman A (2005). InterPro, progress and status in 2005.. Nucleic Acids Res.

[62] Eppig JT, Blake JA, Bult CJ, Kadin JA, Richardson JE (2007). The mouse genome database (MGD): new features facilitating a model system.. Nucleic Acids Res.

[63] Kasprzyk A, Keefe D, Smedley D, London D, Spooner W (2004). EnsMart: a generic system for fast and flexible access to biological data.. Genome Res.

[64] O'Brien KP, Remm M, Sonnhammer EL (2005). Inparanoid: a comprehensive database of eukaryotic orthologs.. Nucleic Acids Res.

[65] Hamosh A, Scott AF, Amberger J, Valle D, McKusick VA (2000). Online Mendelian Inheritance in Man (OMIM).. Hum Mutat.

[66] Wheeler DL, Barrett T, Benson DA, Bryant SH, Canese K (2007). Database resources of the National Center for Biotechnology Information.. Nucleic Acids Res.

