# Supplementary material for: “Guilt by Association” Is the Exception Rather Than the Rule in Gene Networks
Source: PLoS Comput Biol. 2012 Mar 29;8(3):e1002444. doi: 10.1371/journal.pcbi.1002444 (PMC3315453; doi:10.1371/journal.pcbi.1002444)
Supplement: Table S1 — Table showing unusually critical gene ontology groups. All GO groups with 10% or more of their connections exhibiting more than a 0.01 effect upon average precision. The vast majority of GO groups have relatively few connections that might be thought to encode functional information about the group. (DOC) [file pcbi.1002444.s009.doc]

| **GO ID** | **GO description** |
| --- | --- |
| 4549 | 'tRNA-specific ribonuclease activity' |
| 6379 | 'mRNA cleavage' |
| 6378 | 'mRNA polyadenylation' |
| 175 | '3'-5'-exoribonuclease activity' |

**Supplementary Table 1**: Unusually critical gene ontology groups. All GO groups with 10% or more of their connections exhibiting more than a 0.01 effect upon average precision. The vast majority of GO groups have relatively few connections that might be thought to encode functional information about the group.
