# Supplementary material for: “Guilt by Association” Is the Exception Rather Than the Rule in Gene Networks
Source: PLoS Comput Biol. 2012 Mar 29;8(3):e1002444. doi: 10.1371/journal.pcbi.1002444 (PMC3315453; doi:10.1371/journal.pcbi.1002444)
Supplement: Text S1 — Text describing additional experiments and results, including details on cross-validation (section 1), alternatives to “basic GBA” and critical edges (section 2), genetic interaction profile data (section 3), additivity of critical edges effects (section 4), protein complexes (section 5), and pruning networks (section 6). (DOC) [file pcbi.1002444.s010.doc]

# Supplementary information

## Section 1: Precision-recall

An important concept is the use of precision-recall curves for evaluating predictions made with cross-validation. In particular a common metric is the area under a plot of precision against recall, analogous to the even more common use of area under a receiver operating characteristic curve (ROC). The area under the precision-recall curve is equivalent to the average precision and is useful for evaluating prediction performance because it is particularly sensitive to the effects of a single highly-ranked correct guess: correctly giving the top ranking to a true positive has a much stronger impact than the exact rank of other true positives. In contrast, the area under an ROC curve is better at capturing trends in pushing true positives nearer the top of the ranking than expected by chance. In other words, precision-recall rewards a method that can provide one good prediction, with subsequent errors having much less effect on the evaluation. Thus a single edge in the network, connecting two positive examples which are split up by cross-validation, can explain much of the performance for a single learning task when assessed by average precision. It is a measure that maps very conveniently onto experimental requirements since 1/(average precision) represents the number of genes that would need to be tested before a “hit” would be obtained, on average.

## Section 2: Alternatives to “basic GBA” and critical edges

Our results are largely based on the use of a simple GBA algorithm that considers only the direct neighbours of a gene in the predictions (“Basic GBA” or BGBA). This fast algorithm was used to enable the extensive analysis we did, which involved performing millions of analyses, and it also provides a very simple interpretation. Previously we have shown that BGBA performs well compared to other more sophisticated methods , and others have shown that in general performance among methods is strongly correlated, but one might reasonably ask whether our conclusions regarding critical edges depend on this choice. We therefore considered two additional methods that cover a wide range of algorithm classes in use. One class of methods explicitly uses “indirect connections”; the exemplar we chose is GeneMANIA, which does this in a sophisticated way using network label propagation. A second class of methods does not use an explicit network representation, exemplified by the kernel-based support vector machine (SVM) . We note that in both cases there is good reason to think that critical edge effects would hold true. For GeneMANIA, it has already been shown that much of the performance comes from direct connections, so removing single direct connections can still have a large effect on performance.

We repeated some of our key experiments on the aggregated yeast network using these methods. First, we tested whether exceptional connections identified by BGBA remained exceptional regardless of method. To test this, we constructed networks using the 1000 and 2000 most exceptional connections and networks excluding those connections. For SVMs we used the implementation in MatLab using default parameters (a linear kernel). For the SVM, the importance of the 1000 included connections already outweighed the importance of the rest of the network (Average precision of 0.0783 vs average precision of 0.0783), while in the case of GeneMANIA, this point had not been quite reached by 1000 edges included (Average precision 0.0748 vs. 0.1052), but well past by the time 2000 connections were included (Average precision 0.1160 vs. 0.0896). The methods worked well, but again depended on a tiny fraction of connections for their performance. Further, these were the same connections identified by BGBA, despite SVM, in particular, not using a direct “guilt” based approach. We would expect this from our previous findings that most methods essentially use the information encoded by BGBA.

Second, we tested whether the pruning of the network based on node degree altered their performance. In both cases the methods did not yield decreased MAP performance after network pruning, indicating the same network connections are critical regardless of method. We conclude that the issues we exposed using BGBA are not simply an artifact of the method.

## Section 3: Genetic interaction profile data

Our primary analysis of the Constanzo et al. network was based on the representation of the data in BioGRID, in which an edge is assigned to a pair of genes if they genetically interact. An alternative representation, used by Costanzo et al, is profile-based, in which edges are constructed between genes based on the correlation of their genetic interaction profiles. In this representation, from the point of view of a gene, the mutation of another gene is a “condition” in which mutation of the first gene was tested for a phenotype. Thus two genes are linked in the network if they have similar patterns of genetic interactions. Such profile-based methods are also used for gene coexpression and for phylogenetic profiles, but in those cases there is no other way in which to construct the network. For genetic interactions, we can view the “profile” interaction matrix as derived from the “direct” interaction matrix, and indeed it represents “indirect” interactions (two genes in the original network will be strongly connected in the derived network if they share many interaction partners). The reasoning behind the use of the “indirect” matrix rather than the “direct” one is that genetic interactions are between potentially redundant pathways so that indirect associations may involve genes in the same pathway. However, for the purposes of GBA, another motivation may come from the empirical observation that the indirect matrix provides better cross-validation performance. We predicted that this higher performance would come with increased susceptibility to exceptional edge effects.

As expected, constructing the genetic interaction network this way did improve performance (by 32%), but it also increased criticality very substantially, as measured by the effect of node degree pruning, which now increased performance (see below). Importantly, the increase in criticality occurred no matter what threshold was chosen to sparsify the network (or even if the network was analyzed as a fully connected weighted interaction network). Of course, in this case, we do not care if interactions in the profile network are critical. More important is the question of whether the learnable information in the network is critically dependent on connections in the original genetic interactions; that is, to what extent does network performance depend on single observations? In this case, pruning the original network by node degree and then constructing the genetic interaction profile network the same way actually resulted in substantially increased performance, from a MAP of 0.0152 to 0.0195 in the fully connected weighted network, with sparsification of the network not effecting this trend (e.g., choosing a threshold of 0.1 on correlations produced 478798 connections in the profile network and an MAP of 0.0169, while the half-pruned network had an MAP of 0.0222). We conclude that using a profile-based network for analyzing genetic interaction data does not escape the issues of critical edges we showed for other types of networks.

## Section 4: Additivity of critical edge effects

We next move to considering the effect of edges that are not the single most influential for any GO group. We adopt a threshold requiring that the loss of the connection effect at least one GO groups performance by at least 10%, yielding 4870 connections. A natural assumption would be that these critical edges would be exclusively between genes within the set being assessed; that is, connections whose association implies guilt. But it is also possible for associations to correctly imply non-guilt for non-guilty genes, and thus connections between two genes outside of the set can also provide correct (and critical) information, albeit more rarely. In this case, a gene connected to an in-set gene is, itself, not part of the set, but would be highly ranked were it not for a critical edge to a gene outside the set. We assessed the fraction of critical edges (affecting performance by >10%) whose endpoint genes both lie within the gene set. We found that in 50% of critically connected GO groups, the connections were purely internal , but external critical edges do occur in the remainder of cases. and might be expected to play more diverse role across a large fraction of GO groups (since encoding the absence of connectivity).

One concern in relaxing our definition of “critical edge” to “affecting performance by >10%” in this way is that we are overestimating the role of criticality because the critical edges are, in fact, providing the same information. That is, it is possible the loss of two critical edges has no more effect than one. To examine this possibility, we calculated the GO performance resultant from the loss of the top two critical edges and compared it to the “expected” change (which we took to be the sum of the individual effects). We found that in most cases, the effect of removing two critical edges to be identical to the sum of their individual effects; that is, there is no network information beyond the information encoded by the individual critical edges (r~0.68 with the trend line along the identity line). It is possible for two critical edges to have a cooperative effect (e.g., if they both connect to the same gene, removing both will degrade performance by more than either alone); although this tends not to happen since it is form of criticality dependent on removing two connections. Frequently, removing two critical edges has less of an effect than the sum of their effects; this particularly is the case of internal and external critical edges where once an internal edge is lost, the external edge’s encoding not to prioritize another gene ahead of the internally connected gene has less value (and could even now have negative value).

## Section 5: Protein Complexes

In our analyses, we include protein complexes, but it is also possible to regard them as a special case. Recall that we show how protein complexes tend to be very distinct in their performance from other GO groups, and provide no strong generalizability. The independent nature of protein complexes can be made even more explicit by constructing from the original network one which performs well only on protein complexes. This should be possible by leaving densely connected subnetworks intact, but removing other connections. Since many more sophisticated methods exist to determine protein complexes in data, this is not our focus, however, we did perform this network separation using the MINT data. Partitioning the MINT network into connected subnetworks based on the number of length 2 paths between genes (i.e., thresholding the squared adjacency matrix at 10 required connections) produced a network which performed very poorly on non-protein-complexes (Average precision of 0.027), but quite well on protein complexes (Average precision of 0.17). Performing the same analysis on our full network is less simple (since purely separated components are less common given the higher overall density), but would likely not be problematic with a more sophisticated algorithm.

## Section 6: Pruning networks

We showed that a pruned network that privileges low-node-degree edges retains prediction performance. Here we describe some additional control experiments that support the idea that this pruning does not have undesirable side effects. First, while prediction performance may be similar, it is possible that the actual predictions being made are not similar to the original network; that is, the genes being correctly predicted for a given GO group are not the same. To assess this, we compared the rank of gene predictions in the original network, with the rank of gene predictions in the sparser network. The rank of a given prediction in n-fold cross-validation is simply the inverse of its precision. The difference between these precisions for predictions made from the two networks was plotted against the prediction of the original prediction. In essence, this asks whether genes that scored well (or poorly) in the original network are similar in the two networks. The trend for individual GO groups (e.g., Figure S7a) is that genes which scored particularly well or poorly in the original network retain those scores in the new network, while genes with middling scores in the original network are more variable. This is in accordance with the intuition that scoring very well should be consistently significant, but also that scoring very badly is likely significant.

Another potential problem with network pruning might be thought to be that node degree is known to be an important functional correlate and we are altering node degree; in other words, there is a risk of introducing a new artifact. However, this method barely alters the ranking of genes by node degree (r>0.99) because most low node degree connections are to high node degree genes.

## Supplementary references

## 1. Gillis J, Pavlidis P (2011) The role of indirect connections in gene networks in predicting function. Bioinformatics.

## 2. Prasad TS, Kandasamy K, Pandey A (2009) Human Protein Reference Database and Human Proteinpedia as discovery tools for systems biology. Methods Mol Biol 577: 67-79.

## 3. Mostafavi S, Ray D, Warde-Farley D, Grouios C, Morris Q (2008) GeneMANIA: a real-time multiple association network integration algorithm for predicting gene function. Genome Biol 9 Suppl 1: S4.

## 4. Brown MP, Grundy WN, Lin D, Cristianini N, Sugnet CW, et al. (2000) Knowledge-based analysis of microarray gene expression data by using support vector machines. Proc Natl Acad Sci U S A 97: 262-267.

## 5. Lee Y-J, Mangasarian OL (2001) SSVM: A Smooth Support Vector Machine for Classification. Computational Optimization and Applications 20: 5-22.

## 6. Costanzo M, Baryshnikova A, Bellay J, Kim Y, Spear ED, et al. (2010) The genetic landscape of a cell. Science 327: 425-431.

# Supplementary Figure Legends

**Figure S1**. Most GO groups are strongly affected by removing a single connection. Shown is the fraction of average precision performance contributed by a single critical edge for each GO group.

**Figure S2**. Most GO groups in most networks are affected by removing a single connection. For each of our constituent networks, plus the Yeastnet network, we assess the importance of removing each connection in the context of that network. Yeastnet and the genetic interaction network are somewhat outliers due to optimization with respect to GO (Yeastnet) and low performance of direct interactions in the genetic interaction network.

**Figure S3**. GO terms share critical edges. The number of GO groups with their critical edges included rises more rapidly than the number of connections, due to overlap of critical edges; we call such critical edges “exceptional”.

**Figure S4**. Protein complexes have distinctive properties. A) Protein complexes have exceptionally high precision-recalls in GBA B) The density of in-group connections is very high in protein complexes, and uniquely so, so that if a given group (by GO) of genes forms a fully connected sub network, it is assuredly a protein complex. C) Because of their density of in-group connections, protein complexes contribute very strongly to the GO groups not dominated by critical edges, despite their low prevalence.

**Figure S5**. In the yeast network, node degree is a correlate of average criticality of the connections for that gene. For each node degree, the fraction of connections which are critical for that node are shown, and clearly declines with increasing node degree.

**Figure S6**. Heavy-tails are characteristic of the original protein interaction network but not the pruned network. A) The node degree distribution of the original network is shown, as well as the power-law fit, showing the very heavy tail. B) The node-degree distribution of the pruned network is shown, as well as the power-law fit, showing no heavy tail, as well as exhibiting a characteristic node degree to the distribution.

**Figure S7**. Pruning retains significant predictions. A) For a particular GO group, the average precision of individual genes from complete prediction is shown along the X axis, and the similarity of those precisions to those determined in the pruned network is shown along the y-axis, by rank. B) The trend between precisions pre and post pruning is shown averaged across all GO groups, with the black line individuating the mean for a given decile, and the grey lines showing the standard deviation.

**Figure S8**. Figure showing interaction reports are becoming less functional over time. The relationship between the year of an interaction report (by citation in BIOGRID), and the smoothed average functionality (sliding window of 500 points) as encoded by semantic similarity of edges is plotted.
